# Mesenchymal Stem Cell Transplantation Enhancement in Myocardial Infarction Rat Model under Ultrasound Combined with Nitric Oxide Microbubbles

**DOI:** 10.1371/journal.pone.0080186

**Published:** 2013-11-14

**Authors:** Jiayi Tong, Jiandong Ding, Xiangbo Shen, Long Chen, Yeping Bian, Genshan Ma, Yuyu Yao, Fang Yang

**Affiliations:** 1 Institute of Cardiology, Southeast University, Nanjing, Jiangsu Province, China; 2 Jiangsu Province Official Hospital, Nanjing, Jiangsu Province, China; 3 Jiangsu Key Laboratory for Biomaterials and Devices, School of Biological Science and Medical Engineering, Southeast University, Nanjing, Jiangsu Province, China; Rutgers - New Jersey Medical School, United States of America

## Abstract

**Objective:**

This study evaluated the effects of ultrasound combined with the homemade nitric oxide (NO) micro-bubble destruction on the in vitro proliferation, apoptosis, and migration of mesenchymal stem cells (MSCs). Furthermore, we studied whether or not irradiation of the NO micro-bubble combined with bone-marrow derived MSC infusion had a better effect on treating myocardial infarction. The possible mechanism of MSC delivery into the infarcted myocardium was also investigated.

**Methods:**

The murine bone marrow-derived MSCs were isolated, cultured, irradiated, and combined with different concentrations of NO microbubbles. MTT proliferation assay, annexin V-FITC apoptosis detection, migration assay, and RT-PCR were performed 24 h after the irradiation. The NO micro-bubbles was a intravenously injected, followed by the infusion of MSCs, which were labeled by CM-Dil. Myocardium was harvested 48 h later and the distribution of MSCs was observed by laser scanning confocal microscope after frozen sectioning. Echocardiography, histological examination, RT-PCR, and western blotting were performed four weeks after the cell transplantation.

**Results:**

Ultrasound combined with 1:70 NO micro-bubbles had no significant impact on the proliferation or apoptosis of MSCs. Transwell chamber findings demonstrated that MSCs migrated more efficiently in group that underwent ultrasound combined with 1:70 NO micro-bubbles. The Real-time PCR results indicated that the expression of CXCR4 was much higher in the group undergoing ultrasound combined with 1:70 NO micro-bubbles. The normalized fluorescence intensity greatly increased in the group of US+NO micro-bubbles and the cardiac function was also markedly improved. Immunohistochemical staining showed that the capillary density was much greater in the group of US+NO micro-bubbles as compared to that of the other groups. RT-PCR and western blotting also revealed a higher SDF-1 and VEGF expression in the group of US+NO micro-bubbles.

**Conclusions:**

NO micro-bubbles could be used in the cell transplantation, which efficiently promoted the MSC homing into the infarcted myocardium.

## Introduction

Acute myocardial infarction (AMI), as a main presentation of ischemic heart diseases, is a leading cause of death all over the world. Although the therapeutic strategies of pharmacologic intervention, percutaneous coronary intervention, and coronary artery bypass grafting can restore the blood flow and keep the viable myocardium working, these approaches cannot restore the normal cardiac functions as a result of myocardial necrosis. Therefore, it is imperative to develop an effective therapeutic approach to regenerate the myocardium and restore its normal systolic and diastolic functions following AMI. Some recent advances in cellular therapies, such as endothelial progenitor cell (EPC) and mesenchymal stem cell-based strategies, have demonstrated a great potential for the regeneration of ischaemic tissue and the promotion of neovascularization [1-3]. Despite a large body of basic research and ongoing clinical trials, the poor engraftment rate has limited the in vivo reparative capacity of these cells. How to improve the transplantation efficiency is still a remaining problem. 

It has been reported that micro-bubble destruction under ultrasound irradiation has created pores in the capillary walls. This method has been applied for the targeted drug and gene delivery into the arterial vasculature [4]. In 2006, Zen et al. [5] reported that ultrasound combined with albumin micro-bubble destruction greatly enhanced neovascularization by an increase in the bone marrow mononuclear cell endothelial attachment, leading to an improvement in blood perfusion and cardiac function. However, these micro-bubbles were common commercial injectable products without any biological factors. Naturally, the targeted delivery could be subjected to certain restrictions. Nitric oxide (NO) is a very important second messenger and a fundamental factor in cardiovascular homeostasis [6-8]. 

In this study, we intended to develop a new kind of micro-bubble, with NO loaded in its core. We evaluated the effects of destruction on the in vitro proliferation, apoptosis, and migration of the mesenchymal stem cells (MSCs) due to ultrasound combined with NO micro-bubbles. Furthermore, we intended to study whether US irradiating NO micro-bubble destruction combined with bone-marrow derived MSC infusion had a better effect on treating myocardial infarction. Also, the possible mechanisms of the MSC targeted delivery into the infarcted myocardium was investigated.

## Materials and Methods

### Isolation, culture, and phenotype analysis of rat MSCs

The study protocol was complied with the Guide for the Care and Use of Laboratory Animals published by the US National Institutes of Health (NIH Publication No. 85-23, revised 1996). Sprague Dawley (SD) rats were purchased from the Lab Animal Center of Southeast University (Nanjing, China). Bone marrow derived MSCs were isolated as previously described [9]. Briefly, the SD rats were euthanatized and bone marrow from tibias and femurs was flushed with PBS. Mononuclear cells were separated by density-gradient centrifugation with Ficoll-Paque™ (Amersham Biosciences, Uppsala Sweden). Cells were resuspended in DMEM (Gibco, USA) supplemented with 10% fetal bovine serum (FBS) and antibiotics. Non-adherent cells were removed after 48 hours, replacing the media every two to three days. The passaged cells were cultured by using standard protocols. An MSC passage was chosen from among the 3rd to 5th passage for all experimental use. The morphological features and characteristic surface makers detected by flow cytometry were used to identify the MSCs as reported before.

### The preparation of NO microbubbles

Since NO is unstable under general situation, we wrapped it into the micro-bubbles. 1,2–Dipalmitoyl–sn–Glycero–3–phosphoethanolamine–N-[Methoxy (Polyethylene glycol)-2000] (DPPE-PEG2k) was purchased from Avanti Polar Lipids, Inc. (Alabaster, AL). The L-α-phosphatidylcholine (PC, lyophilized powder) was purchased from Sigma-Aldrich, Inc. NO and all the other reagents were analytical grade and were used as received. PC/DPPE-PEG2k (95:5, molar ratio) was dissolved in chloroform in a round-bottom flask. Chloroform was removed under a vacuum evaporation until the thin film formed. A phosphate buffer solution (PBS, pH: 7.4±0.1) was added to the dried lipid thin films to create a lipid concentration of 1 mg/mL. The lipid suspension was then mixed well above the phase transition temperature of the lipids (60°C) to form a milky solution of multilamellar liposomes. Under the anaerobic condition, the multilamellar liposome suspension was continuously sonicated with a probe at 100 W with constant purging using a steady (4 mL/min) stream of NO gas for 5 min to form the lipid-encapsulated NO micro-bubbles. The mean diameter of the bubbles was analyzed using N4 PLUS Submicron Particle Size Analyzer (Beckman Coulter, USA). Milli-Q pure water was used as the blank solution, where the sample was added until an appropriate concentration was indicated. Each sample was tested in triplicate. Samples in solution were placed between the glass slides and observed with an Axioskop 40 microscope equipped with a Coolsnap MP3.3 camera (Carl Zeiss, Germany).

### Assay effects of ultrasound combined with NO microbubbles on the proliferation and apoptosis of MSCs

The experiments were divided into two groups, including: the blank control group and the ultrasound combined with NO micro-bubble group. The NO micro-bubble group contained 1:20, 1:50, and 1:70 subgroups according to different concentrations of NO micro-bubbles. MSCs were seeded at a density of 5×10^5^ cells per well, every other well in the 96-well culture plates. The 96-well culture plate was then placed on the surface of the double distilled water at the temperature of 37°C. The ultrasonic probe was vertically placed about 8 cm to the top of the culture plates and continuously irradiated for 60 seconds (1 MHz, 1 W/cm^2^). After ultrasonic intervention, the cells were further incubated for 24 h and the proliferation and apoptosis were assessed according to the following protocols.

1 Assessment of cell proliferation ability was performed according to the MTT assay. The treatment method for the MSCs in each group was the same as what was described above and each group had eight parallel wells. At the culture period, the cells were incubated with the MTT solution (Sigma, USA) for 4 h. The medium was then removed and formazan salts were dissolved with 150 µL of dimethylsulfoxide (DMSO, Sigma, USA). Finally, the absorbance was measured at 490 nm with the Model 680 Microplate Reader (Bio-Rad, American).
2 Cell apoptosis was measured by Annexin V-FITC Apoptosis Detection Kit (Biovison, USA). Cells were resuspended in 500 µL of 1× Binding Buffer. A total of 5 µL of Annexin V-FITC and 5 µL of propidium iodide (PI 50 mg/ml, optional) were also added, followed by a 5-min incubation at room temperature in the dark. The Annexin V-FITC binding was analyzed by flow cytometry (Ex=488 nm; Em=530 nm) using FITC signal detector. The PI staining was analyzed by the phycoerythrin emission signal detector.

### Assay effects of ultrasound combined with NO microbubbles on the migration of MSCs

MSCs migration assay was utilized by a modified Transwell chamber (Corning Costar, Cambridge, MA). The cells were seeded in the upper chamber at a density of 1×10^5^ cells/mL. A total of 500 μL of the medium with 30 ng/mL SDF-1 (PeproTech, Rocky Hill, NJ) was added into the lower compartment. The cell experiments were then divided into four groups: control, ultrasound, ultrasound combined with commercial micro-bubbles, and ultrasound combined with NO micro-bubble groups. Commercial micro-bubbles were added to the ultrasound combined with commercial microbubbles group with a suitable concentration. The NO microbubbles were added to the ultrasound combined with NO microbubbles group with a suitable concentration (1:70). Also, equal amount of PBS was added to the control and ultrasound groups. The ultrasonic radiation (1MHz, 1 W/cm^2^) was started for 60 s for the other three groups except for the control group. They were then incubated for 24 hours in 5% CO_2_ at 37°C. The cells without any migration were removed from the upper surface of the filters using cotton swabs and those that migrated to the lower surface of the filters were fixed in methanol, followed by Crystal Violet staining. Migration was determined by counting the cell numbers with a microscope at 100× magnification. Five visual fields were randomly chosen for each assay. The average number of the migrating cells in five fields was taken as the cell migration number of the group. Experiments were performed in sets of four for each group.

### Real-time fluorescence quantitative Polymerase Chain Reaction for CXCR4 expression

After migration, total RNA was isolated from the MSCs using Trizol Reagent (Invitrogen, USA) following the manufacturer’s instruction. First strand cDNA synthesis was performed using an MBI RevertAid First Strand cDNA Synthesis Kit (MBI, Lithuania). Cycle conditions were as follows: 94°C for 5 min followed by 30 cycles (94°C denaturation for 30 s, 50°C annealing for 30 s, and 72°C extension for 30 s), with a final incubation at 72°C for 10 min. The following primers were employed: CXCR4 sense: GCTGAGGAGCATGACAGACA and CXCR4 antisense: GATGAAGGCCAGGATGAGAA; β-actin sense: TGTCACCAACTGGGACGATA and β-actin antisense: GGGGTGTTGAAGGTCT. The mRNA levels were normalized by β-actin housekeeping gene. The Real-time PCR was performed with an iQ5 Multicolor Real-Time PCR Detection System (Bio-Rad Laboratories, Hercules, CA, USA), using SYBR-Green Master Mix (TOYOBO), Japan) in the 25-μL reaction mixtures. All the experiments were repeated six times.

### Myocardial infarction (MI) model establishment and allogeneic stem cell implantation

This study was carried out in a strict accordance with the recommendations in the Guide for the Care and Use of Laboratory Animals (NIH 1996). The protocol was approved by the Committee on the Ethics of Animal Experiments of Southeast University. All the surgeries were performed under the sodium pentobarbital anesthesia and all efforts were made to minimize the suffering. 

The eight-week-old Sprague Dawley (SD) rats were implemented with an abdominal anaesthesia by 2% pentobarbital natrium (40 mg/kg). The airway was maintained by endotracheal intubation. Myocardial infarction (MI) model was induced by ligation of the left anterior descending coronary artery (LAD). A left thoracotomy was performed and the LAD was ligated at a level immediately below the bottom of the left atrium by irreversible tightening of a 6–0 suture loop [10]. The bottom of the left atrium was used as a demarcation point to ensure a consistent placement of the ligature and resultant reproducibility of similar infarct sizes among the groups of animals. A successful performance of coronary occlusion was confirmed by regional cyanosis of the myocardial surface distal to the suture, accompanied by a typical S-T segment elevation on the electrocardiogram (ECG). One week later, the 42 MI rats were alive. They were randomly divided into four groups for cell therapy, including: US+NO microbubbles combined with MSCs infusion group (group 1, n=12), US+commercial microbubbles combined with MSCs infusion group (group 2, n=10), MSCs (1×10^7^ cells suspended in 2 ml PBS) transplanted by the caudal vein injection group (group 3, n=10), the PBS (2 ml) infusion without MSCs transplantation group was set as control group (group 4, n=10). The MI rats of group 1 and 2 were irradiated with a therapeutic US system at the frequency of 1 MHz, and the ultrasonic intensity was set at 1 W/cm^2^. Ultrasound probe was put to the anterior chest for 10 min after the intravenous injection of microbubbles, followed by infusion of MSCs. All the MSCs were labeled by CM-Dil (Introvigen, C7000, USA) before being transplanted. After 48 h of cell transplantation, four rats were sacrificed in each group and hearts were immediately harvested. The survival of implanted cells were identified by the number of CM-Dil-positive cells in the frozen sections (8 μm in thickness) made from hearts of MI under laser scanning confocal microscope, and fluorescence intensity was used to reflect the transplanted cell numbers. The fluorescence intensity of CM-Dil-positive cells was evaluated by biological fluorescence image analysis software (Olympus Fluoview Ver.2.1a). 

### Echocardiographic study

Transthoracic echocardiographic images of the hearts from the rest of the 26 rats were obtained on 4th week post-cells transplantation using an ultra high-resolution ultrasound scanner (Vevo 2100 VisualSonics, Canada) under nembutal anesthesia (the depth of anesthesia were almost the sane). For the M-mode recordings, the parasternal short-axis view was used to image the heart in two dimensions at the level of the papillary muscles. The LV ejection fraction (EF) was recorded along with LV cavity dimensions (end-diastolic and end-systolic).

### Determination of capillary density

All the rats were sacrificed after the completion of echocardiography, and the hearts were removed and paraffin-embedded sections were made to study capillary density. The sections were stained for capillary density using CD-31 primary antibody (Santa cruz) and bounded antibody was detected with Vectastain ABC kit (Vector Laboratories, Burlingame CA) and visualized with DAB (Sigma). The 40× magnified pictures were captured and used for CD-31 counting. For the quantitative measurement, the number of CD-31-positive cells was counted on the infarct border area from the endocardium through epicardium of the midportion of the left ventricular free wall. Five non-overlapping 400× magnification fields from four sections of each heart were randomly selected. The capillary density (CD) was averaged and expressed as the number of capillaries per unit area, and the data were finally statistically analyzed with SPSS.

### RT-PCR and Western blotting

Tissues of the MI area were harvested and minced until the tissues were fully grinded. Total RNA was extracted by using the Trizol system (Invitrogen, USA) according to the manufacturer's guidelines. It was then reverse-transcribed into cDNA processed by the RevertAid™ First Strand cDNA Synthesis Kit (Fermentas, Canada) and the addition of 25 μL of SYBR Green (TOYOBO, Japan) to the reaction system. Rat SDF-1 was amplified with the sense primer of 5’-CCCTGCCGATTCTTTGAG-3 and the anti-sense primer of 5’-GCTTTTCAGCCTTGCAACA-3’. The rat VEGF was amplified with the sense primer of 5’-GCAGCTTGAGTTAAACGAACG-3’ and the anti-sense primer of 5’-AGTTCCCGAAACCCTGAG-3’. Furthermore, β-actin was also amplified with the sense primer of 5’-GCAGAAGGAGATTACTGCCCT-3’ and the anti-sense primer of 5’-GCTGATCCACATCTGCTGGAA -3’ as a housekeeping gene to normalize the expression target gene. All the primers were used at a final concentration of 0.4 μmol/L. The thermal cycling conditions were as follows: 30 seconds at 95°C for pre-denaturation, 30 cycles for 15 seconds at 95°C for denaturation, 1 minute at 59°C for annealing, and 10 seconds at 72°C for elongation. At the end of each cycle, the fluorescence emitted by the SYBR Green was measured. The relative gene expression was analyzed by the 2-DDCt method. All the experiments were repeated six times.

The tissues from the MI area were harvested and minced. Western analysis was carried out using the following primary antibodies: SDF-1 (1:500, Boster, China) and VEGF (1:400, Boster, China). After an extensive washing, the immunocomplexes were detected with horseradish peroxidase-conjugated appropriate secondary antibodies followed by enhanced chemiluminescence reaction and the relative expression of SDF-1 and VEGF were analyzed by one image analysis software.

### Statistical analyses

All the values are presented as mean±standard deviation. Results were compared by one-way ANOVA and Student Newman Keuls test. Statistical analysis was performed using SPSS 18 software. A value of p<0.05 was considered to be statistically significant.

## Results

### The characteristics of MSCs

**Figure 1:**
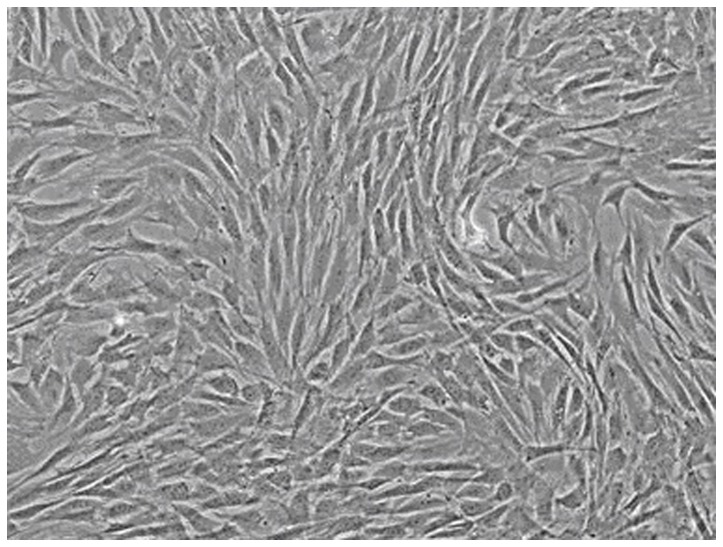
The morphology observation of MSCs under the optical microscopy.

Density gradient centrifugation culture method was used in the isolation and cultivation of MSCs. The morphological features were observed and the characteristic surface makers were detected by flow cytometry. Most of the cultured MSCs were spindle shaped and became more uniform after several passages (Figure 1). These expanded MSCs were uniformly positive for CD44 (99.34%) and CD29 (99.35%), and negative for CD34 (1.47%) and CD45 (1.56%).

### The characteristics of NO microbubbles

**Figure 2:**
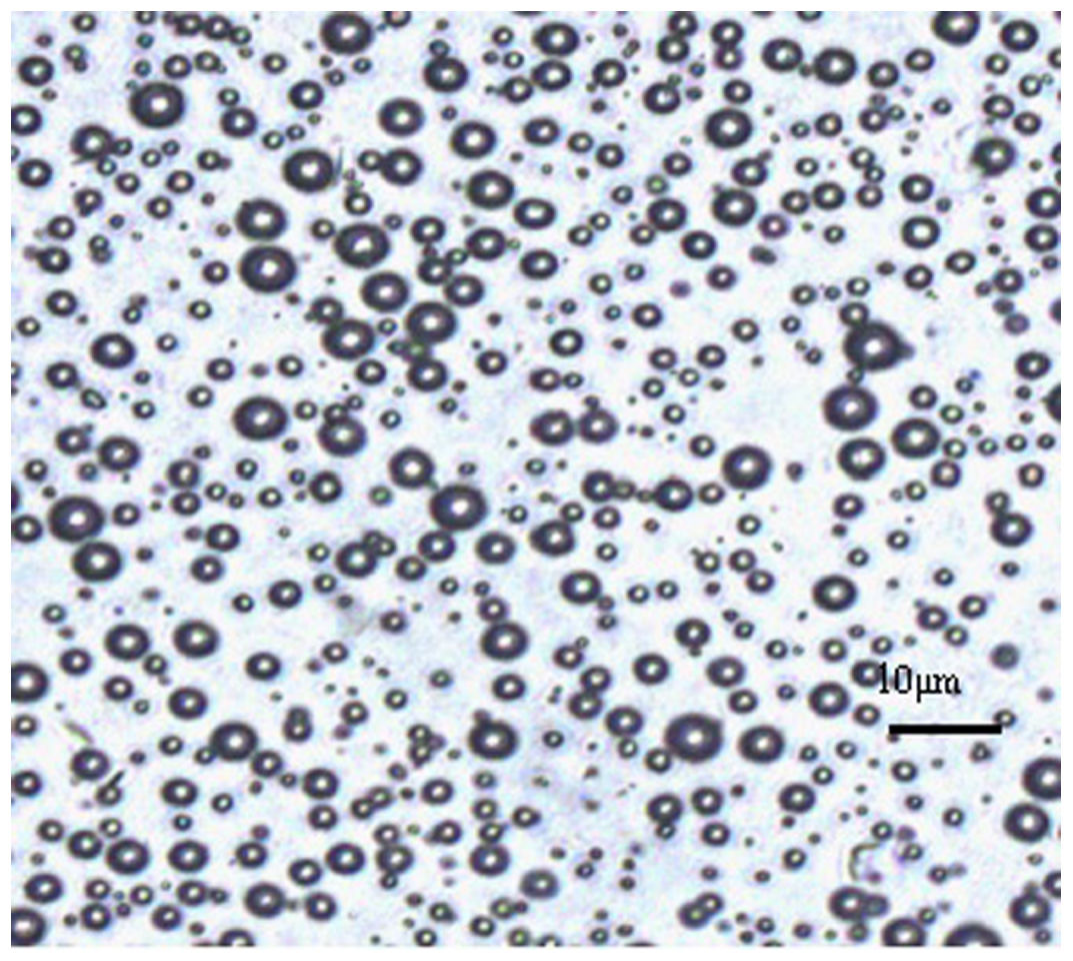
The morphology observation of NO microbubbles under the optical microscopy.

The average mean size of the NO microbubbles was found to be 3.85 μm with a polydisperity index of 0.699. The NO concentration was about 1.33×10^-9^ mmol per microbubble. The microbubble concentration was about 6-8×10^6^ per milliliter. The microscopic images under the light microscopic observation indicated the regular spherical morphology as shown in Figure 2.

### Effects of ultrasound combined with NO microbubbles on the proliferation and apoptosis of MSCs

The MTT assay was used to assess the proliferation of MSCs, which were treated by the ultrasound combined with NO microbubbles. The OD values were obtained using a reading plate at 490 nm. The relative growth rates were 0.94±0.06, 0.94±0.05, and 0.67±0.04 in the ultrasound combined with 1:70 NO microbubbles group (NO (1:70)), ultrasound combined with 1:50 NO microbubbles group (NO (1:50)), and ultrasound combined with 1:20 NO microbubbles group (NO (1:20)), respectively. We found that the proliferation of MSC was significantly decreased in the ultrasound combined with 1:20 NO microbubble group (p<0.05), but there was no significant difference in the ultrasound combined with 1:70 NO microbubbles group, ultrasound combined with 1:50 NO microbubble group, and the control group. Ultrasound combined with 1:70 NO microbubbles did not significantly inhibit cell proliferation as assessed by the MTT assay.

The representative apoptosis of the MSC was obtained from flow cytometry. The mean percentage of MSC apoptosis were 2.9±1.1%, 3.4±0.9%, 7.9±1.8%, and 19.8±2.9% in the control group (Control), ultrasound combined with 1:70 NO microbubbles group (NO (1:70)), ultrasound combined with 1:50 NO microbubbles group (NO (1:50)), and ultrasound combined with 1:20 NO microbubbles group (NO (1:20)), respectively. The percentage of the apoptotic cells was significantly higher in the ultrasound combined with 1:50 NO microbubbles group and ultrasound combined with 1:20 NO microbubbles group in comparison with the control group (p<0.05). However, there was no significant difference between the ultrasound combined with 1:70 NO microbubbles group and control group. Ultrasound combined with 1:70 NO microbubbles did not significantly increase the MSC apoptosis.

### Effects of ultrasound combined with NO microbubbles on the migration of MSCs

**Figure 3:**
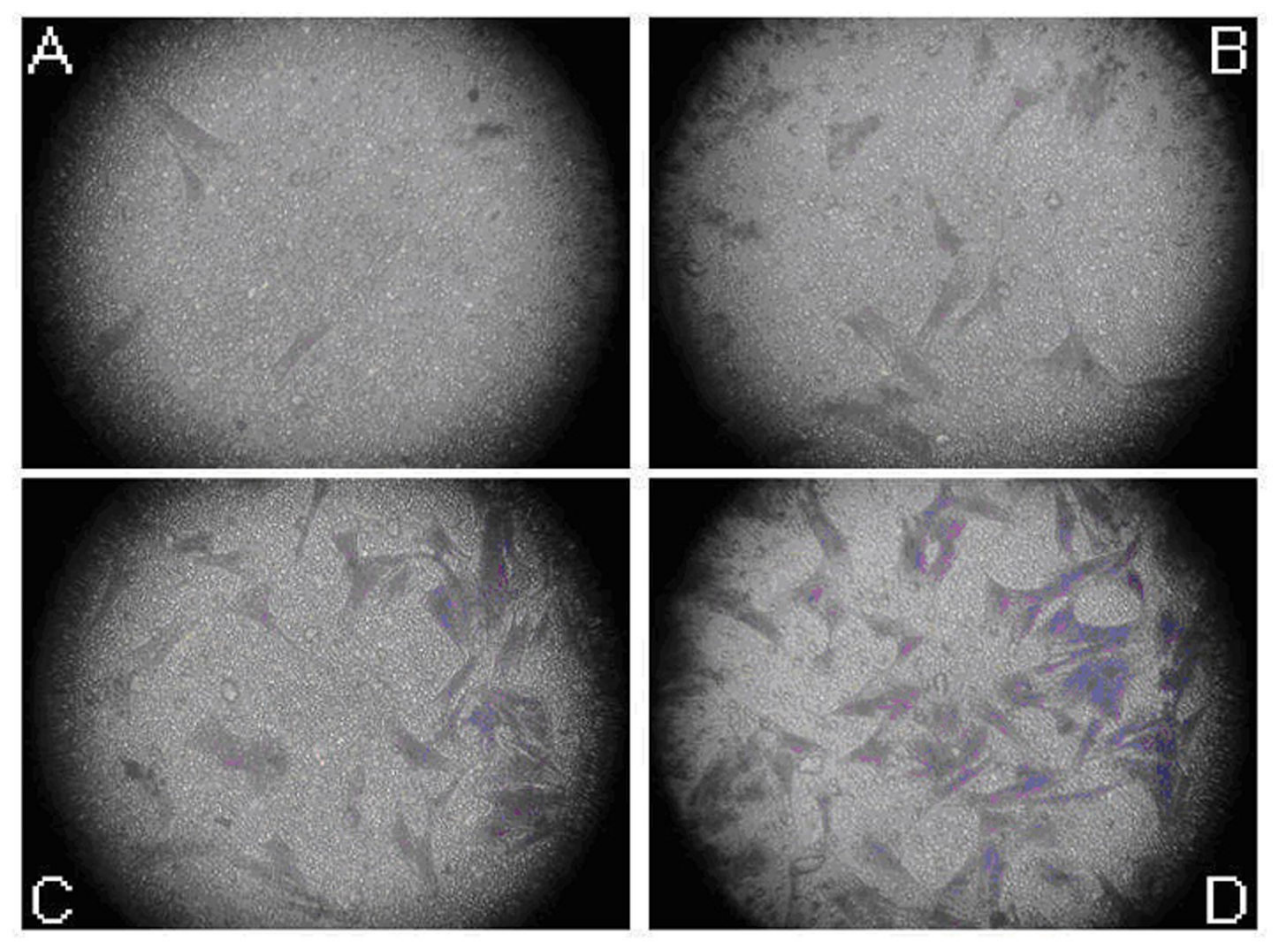
Effects of ultrasound combined with NO microbubbles on the migration of MSCs. Crystal Violet staining was performed to determine the number of migrated cells. (A) Image of MSCs, which migrated to the bottom of the wells in the control group. (B) Image of MSCs, which migrated to the bottom of the wells in the ultrasound only group. (C) Image of MSCs, which migrated to the bottom of the wells in the ultrasound combined with the commercial microbubbles group. (D) Image of MSCs, which migrated to the bottom of the wells in the ultrasound combined with NO microbubbles group. The mean number of migrated MSCs was 58.5±7.47, 32.71±7.10, 19.44±7.21, and 4.80±2.52, respectively. Ultrasound combined with NO microbubbles increased the number of migrated MSCs (p<0.05).

As shown above, the ultrasound combined with 1:70 NO microbubbles were relatively safe for the MSCs. Therefore, the consternation of 1:70 was chosen to verify the role of ultrasound combined with NO microbubbles in the MSCs’ migration. We performed a Transwell migration assay, which was routinely used to study cell migration in response to specific signal stimuli. As shown in Figure 3, the MSCs treated with ultrasound combined with NO microbubbles migrated more efficiently as compared to the other groups (p<0.05).

Crystal Violet staining was performed to determine the number of migrated cells. Figure 3 (A) demonstrates the image of the MSCs, which migrated to the bottom of the wells in the control group. Figure 3 (B) is the image of the MSCs, which migrated to the bottom of the wells in the ultrasound only group. Figure 3 (C) is the image of the MSCs, which migrated to the bottom of the wells in the ultrasound combined with commercial microbubbles group. Figure 3 (D) is the image of the MSCs, which migrated to the bottom of the wells in the ultrasound combined with NO microbubbles group. The mean number of the migrated MSCs were 58.5±7.47, 32.71±7.10, 19.44±7.21, and 4.80±2.52, respectively. Ultrasound combined with NO microbubbles increased the number of migrated MSCs (p<0.05).

### Effects of ultrasound combined with NO microbubbles on the expression of CXCR4

**Figure 4:**
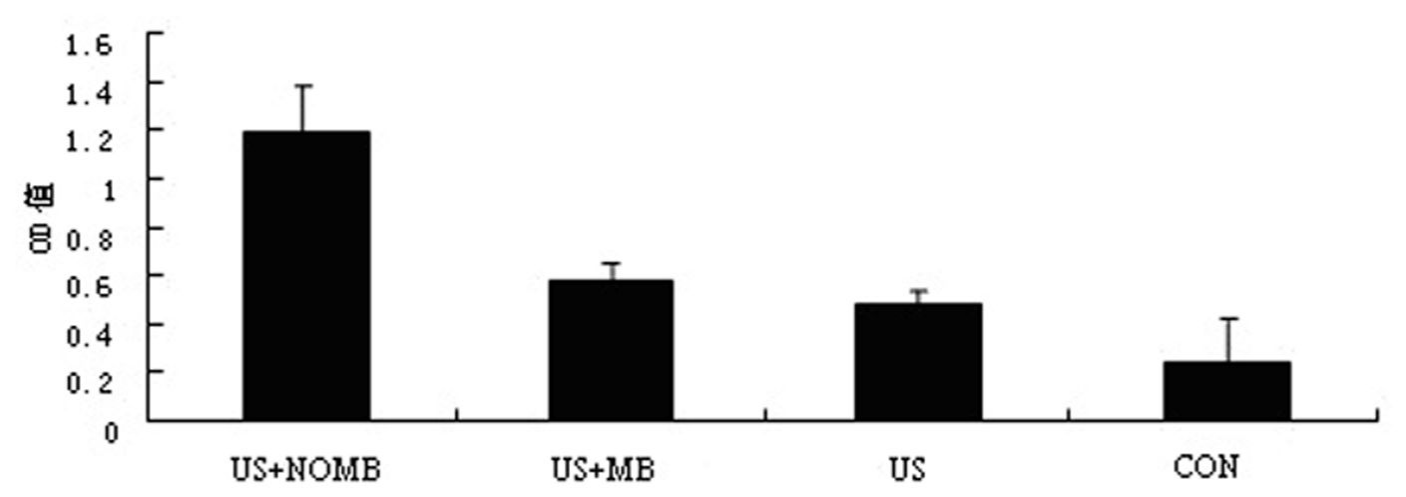
Effects of ultrasound combined with NO microbubbles on the expression of CXCR4 on MSCs. A: The image of group 1, B: The image of group 2, C: The image of group 3, and D: The image of group 4.

Quantitative Real-time PCR was used to assess effects of ultrasound combined with NO microbubbles on the expression of CXCR4 in MSCs. As shown in Figure 4, CXCR4 expression was significantly higher in the ultrasound combined with NO microbubbles group as compared to the other groups (p<0.05).

### Identification of engrafted MSCs

**Figure 5:**
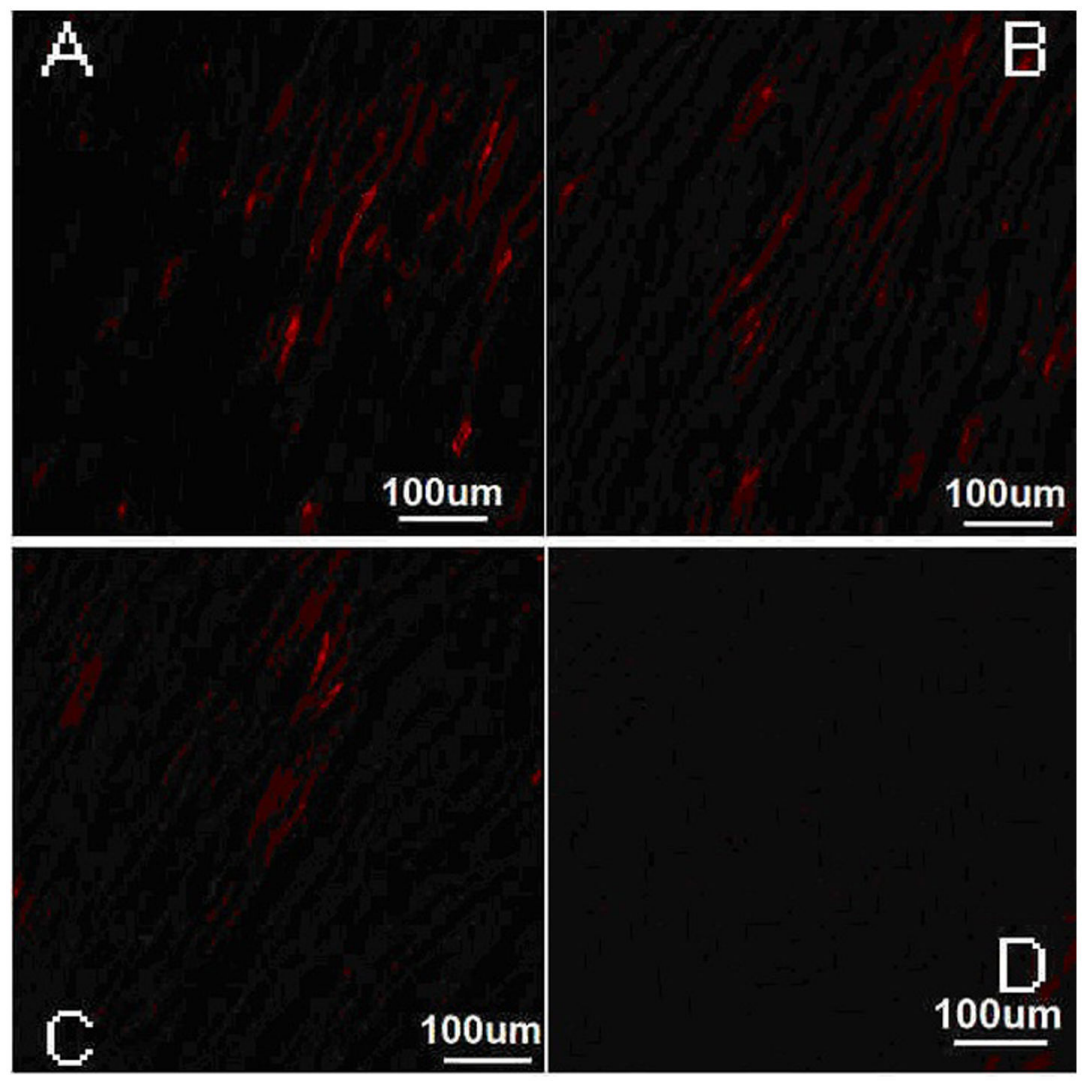
Distribution of MSCs in four groups under the laser scanning confocal microscope. A: The image of group 1, B: The image of group 2, C: The image of group 3, and D: The image of group 4.

All the cultured MSCs could be labeled with CM-Dil, and CM-Dil positive cells were stained in phospholipid bilayer membranes in red under laser scanning confocal microscope (Figure 5(A-D)). The CM-Dil positive cells were located in the infarcted and border areas, while there were nearly none in the normal myocardium. The mean fluorescence intensity was 3140.2±317.1, 2408.9±276.3, 1084.1±241.7, and 617.5±209.3, respectively, from picture A to D (Table 1). Quantification analysis showed that there was a significant difference between group 1 and the other three groups (P<0.05).

**Table 1:** Mean fluorescence intensity in all groups.

**Group**	**Mean fluorescence intensity**	S-N-K Test^*^
1234	3140.2±317.12408.9±276.31084.1±241.7617.5±209.3	ABCD

* Different letters indicate statistical differences in the pair-wise comparison.

### Improvement in cardiac function

**Figure 6:**
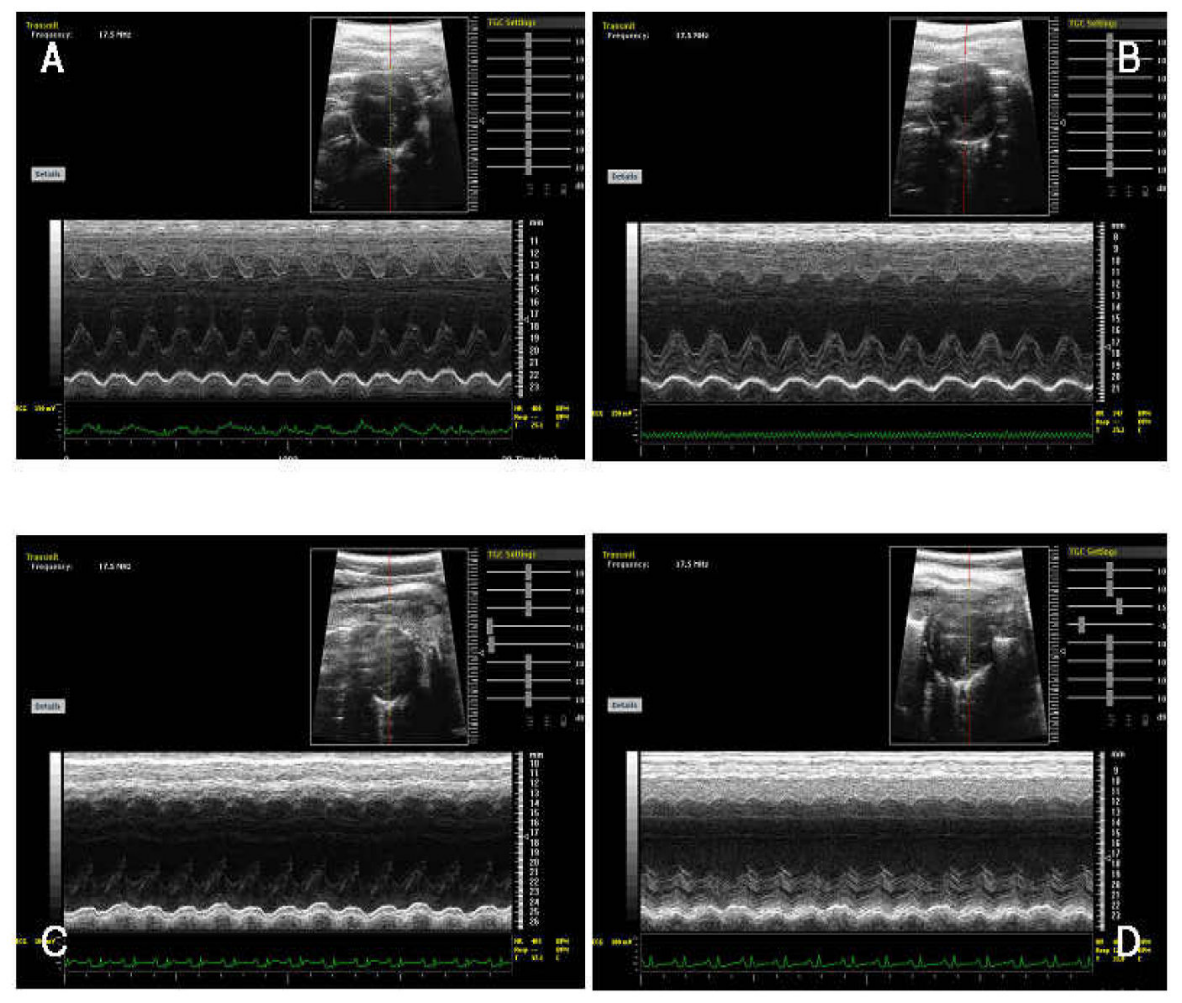
Cardiac systolic function was assessed with the M-mode echocardiography. The ejection fraction (EF) were detected in group 1 (A), group 2 (B), group 3 (C), and group 4 (D).

After the MSC transplantation, the left ventricle systolic function was improved and there was also a significant EF difference between group 1 and 2 (61.27±4.69% and 53.35±3.29%; P<0.05). The same trend was observed for group 1 and 3 (61.27±4.69% and 45.91±4.57%; P<0.05], and group 2 and 4 (61.27±4.69% and 44.16±5.72%; P<0.050. Figure 6 (A- D) showed that EF% was higher in group 1 as compared to that of the group 2, group 3, and group 4. All the p values were less than 0.05. Table 2 shows the S-N-K Test for the four groups.

**Table 2:** Ejection fraction in all groups.

**Group**	**M-EF(%)**	S-N-K Test*
1234	61.27±4.6953.35±3.2945.91±4.5744.16±5.72	ABCC

* Different letters indicate statistical differences in the pair-wise comparison.

### Immunohistochemical analysis of capillary density

**Figure 7:**
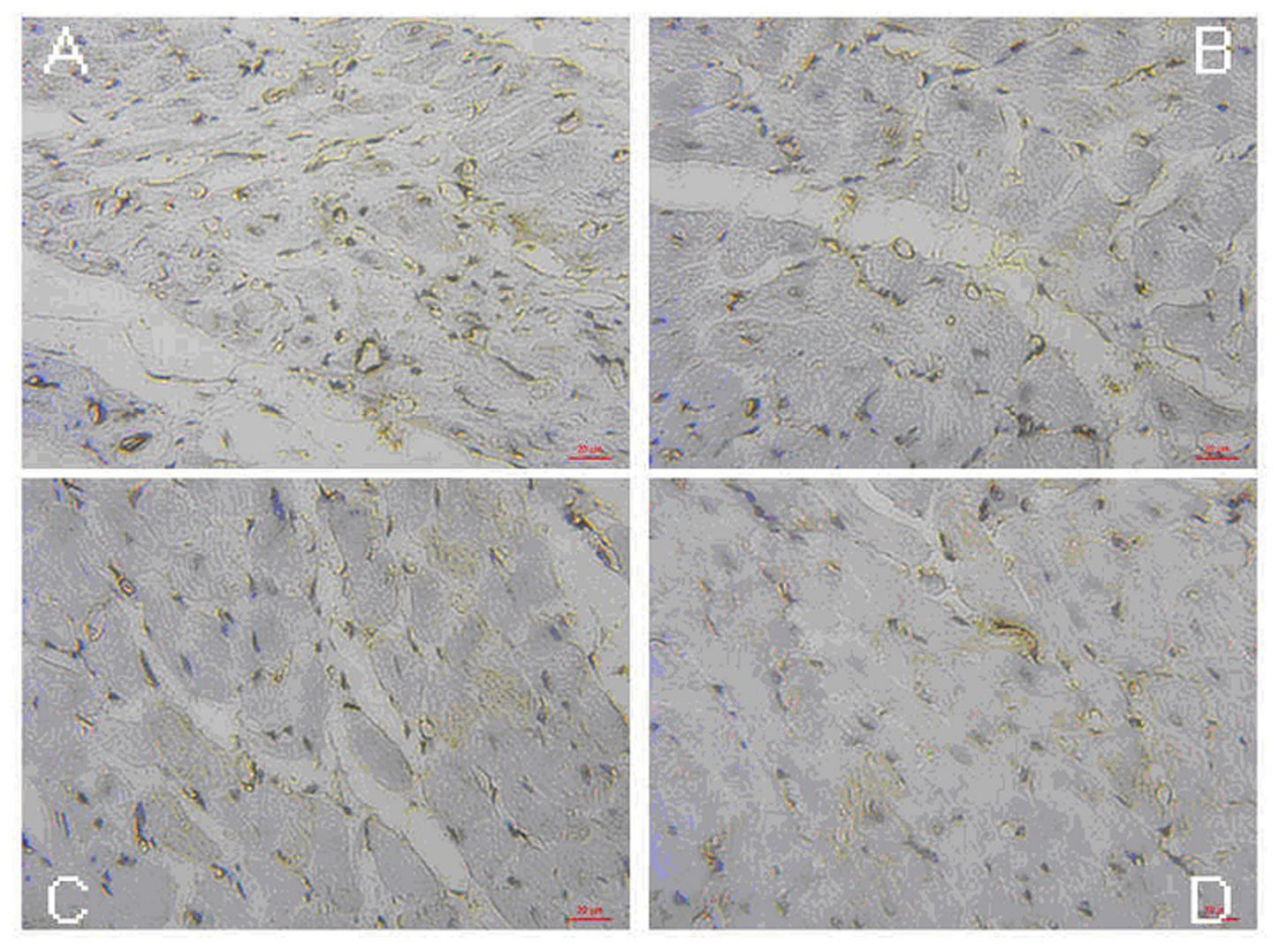
Quantitative analysis of the capillary density in the peri-infarct area 28 days after the MSC transplantation (400×). A: The image of group 1, B: The image of group 2, C: The image of group 3, and D: The image of group 4.

Four weeks after the MSC delivery, we assessed whether NO microbubbles had an effect on enhancing neovasculature formation. Indeed, quantification of capillaries revealed that group 1 had a significantly higher capillary density (46±6) as compared to that of the group 2 (34±5) (p<0.05), group 3 (22±4) (p<0.05), and group 4 (18±8) (p<0.05) (Figure 7A-D, Table 3). This finding indicated that ultrasound combined with NO microbubbles mediated the MSC transplantation, which could augment the capillary density as a beneficial effect for myocardial repair.

**Table 3:** Capillary density in all groups.

**Group**	**Capillary density**	S-N-K Test*
1234	46 ± 634 ± 522 ± 418 ± 8	ABCC

* Different letters indicate statistical differences in the pair-wise comparison.

### Expression of SDF-1 and VEGF with RT-PCR and Western blotting

**Figure 8:**
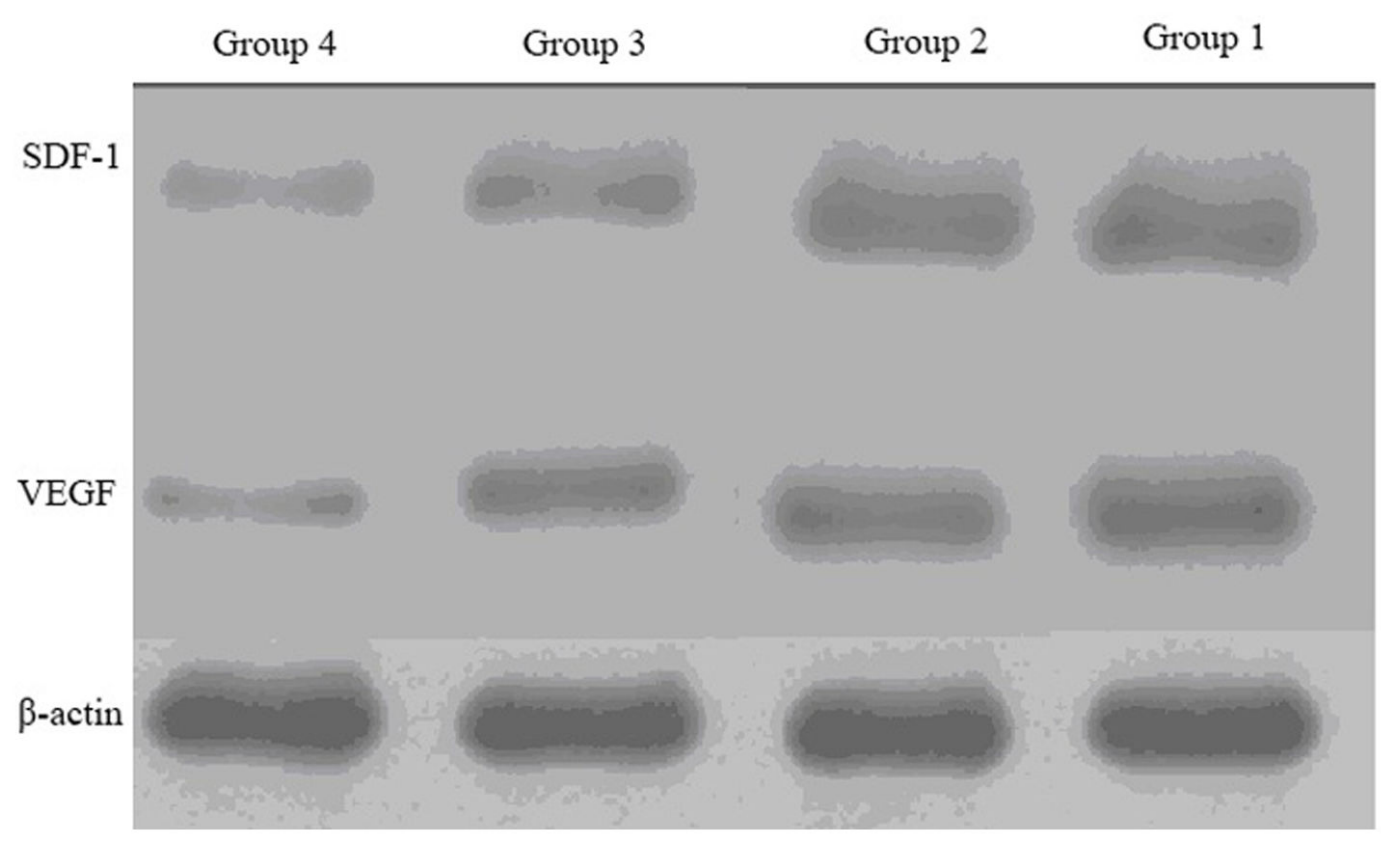
Protein expression of SDF-1 and VEGF determined by Western blotting of the ischemic heart from four groups.

RT-PCR results showed that the level of SDF-1 and VEGF (Table 4) in the infarcted zone was higher in group 1 as compared to that of the group 2, 3, and 4 (all the P values were less than 0.05). Western blotting results showed that the level of SDF-1 (Figure 8, Table 4) in the infarcted zone was higher in group 1 as compared to that of the group 3 and 4 (the P values were both less than 0.05). However, there was no statistical differences between group 1 and 2 (P>0.05). Western blotting results showed that the level of VEGF (Figure 8, Table 4) in the infarcted zone was higher in group 1 as compared to that of the group 2, 3, and 4 (all the P values were less than 0.05).

**Table 4:** The relative expression of SDF-1 and VEGF in all groups.

**Group**	**SDF-1**		**VEGF**	
	**RT-PCR**	**Western blotting**	**RT-PCR**	**Western blotting**
1234	5.74±0.512.15±0.171.87±0.201.03±0.11	0.62±0.0340.59±0.0400.38±0.0190.13±0.009	7.41±0.432.91±0.272.28±0.291.04±0.08	0.67±0.0240.54±0.0110.44±0.0200.12±0.009

## Discussion

Myocardial infarction is characterized by extensive necrosis of cardiomyocytes, which causes irreversible deterioration of cardiac function. Recent researches have shown that the transplantation of stem cells for the repair of myocardial necrosis is a safe and effective method for the treatment of ischemic cardiomyopathy [11]. Because of the ability of the self-renewal and multiple differentiation potential, the MSC becomes good candidates for experimental and clinical applications in stem cell therapy of acute myocardial infarction [12]. However, poor cell viability associated with transplantation has limited the reparative capacity of these cells in vivo. How to improve the transplantation efficiency is still a remaining question.

Microbubbles were originally developed as contrast agents for ultrasonic imaging and diagnosis [13]. Recently, the therapeutic use of microbubbles has become an emerging methodology [14,15] with a high potential for the enhanced directed therapeutic gene, drug, bioactive gas, and stem cell delivery [16]. In these studies, commercial and homemade microbubbles were mostly composed of low-solubility gases, such as SF_6_ or C_3_F_8,_ and a coating of biodegradable material, such as albumin, phospholipids, or polymers. The gaseous free radical NO is an important regulator of a variety of biological functions, especially in the cardiovascular system [17]. However, it can be easily oxidized to nitrate or nitrite under general situation due to its short half-life of only 3-5 s, which could greatly influence its application in the clinical and experimental study. Because of the fact that microbubbles could be stabilized as a new type of biological vector, we wrapped NO into the microbubbles. The novel NO microbubbles could burst to release NO into the targeted tissues. As shown in the results, we have successfully fabricated the NO microbubbles, with the mean size of 3.85 μm (1.33×10^-9^ mmol NO in each microbubble).

The previous studies have demonstrated the safety of the commercial microbubbles [18]. However, there have been no studies about these novel NO microbubbles. Our pervious study indicated that ultrasound with low intensity 0.25 W/cm^2^ and frequency of 1 MHz had no adverse effect on the proliferation, apoptosis, and cell cycle of the endothelial progenitor cells. So we applied the same ultrasonic parameters in this study. The results demonstrated that ultrasound (1 W/cm^2^, 1MHz) combined with a suitable concentration of NO microbubbles (1:70) had no significant influence on the proliferation and apoptosis of MSCs, which was relatively safe to MSCs in vitro and provided more evidence for further studies in vivo.

For the statement mentioned above, we made an innovative use of the ultrasound mediated NO microbubble destruction to promote the MSC homing for the therapy of myocardial infarction. The previous studies have demonstrated that the ultrasound combined with microbubbles could enhance the effect of the cell transplantation in MI treatment [19], but there was no report about the NO microbubble. In our studies, we found that the number of CM-Dil positive cells in the MI area of the US+NO Microbubbles+MSC group was significantly larger than that of the US+Microbubbles+MSCs group, the MSCs group, and the control group. This observation showed that the ultrasound combined with NO microbubble delivery system might have a higher efficiency of prompting the homing and gathering of MSCs to the area of MI. It is well known that SDF-1/CXCR4 plays a very important role in stem cell homing [20], so we made a much more comprehensive study of its mechanism both in vivo and in vitro. In the in vitro experiments, we found that the MSCs under ultrasound combined with NO microbubbles migrated more efficiently and the RT-PCR results revealed that the CXCR4 expression was higher in the US+NO microbubble group. In the in vivo experiments, higher expression of SDF-1 was found in the US+NO Microbubbles+MSCs group as compared to that of the US+Microbubbles+MSCs group, the MSCs group, and the control group. The high expression of SDF-1 and CXCR4 might have been involved in the enhanced attachment of transfused MSCs onto the targeted area. Furthermore, Li et al. [21] reported that the myocardial permeability was improved in the ultrasound irradiation area, which facilitated the homing and gathering of the MSCs into the MI area.

From the echocardiography, we found that the post-infarction left ventricular function improved most in the US+NO microbubbles+MSCs group as compared to the other three groups. It was also reported that the improvement of the left ventricular function was mainly due to the neovascularization and inhibition in fibrosis and LV remodeling. Among these factors, neovascularization was considered to be one of the most important mechanisms of the MI recovery. It could improve the myocardium perfusion and preserved the stunned cardiomyocytes and surviving myocytes from the post-ischemic apoptosis. VEGF has been known to be a multifunctional cytokine that contributes to angiogenesis by both direct and indirect mechanisms. It is reported to be a major contributor to angiogenesis by increasing the number of new capillaries. After myocardial infarction, ischemia and hypoxia stimulates the VEGF autocrine activity. Song et al. [22] have reported that the therapeutic ultrasound-mediated microbubble cavitation induced the VEGF secretion and angiogenesis in the normal skeletal muscles. Furthermore, in this study, we used ultrasound combined with NO microbubbles infusion to mediate the MSC delivery. Compared with the US+microbubbles+MSCs group, the MSCs group, and the control group, the US+NO microbubbles+MSCs group revealed a remarkable up-regulation in the VEGF expression. At the same time, the capillary density was also increased.

In summary, our study has demonstrated that an intravenous infusion of MSCs combined with US+NO microbubbles enhanced the targeted delivery of the MSCs into the infarcted myocardium and induced the regional angiogenic response. It was beneficial for an improvement of LV perfusion and cardiac function of the ischemic heart. These findings suggested that this novel cell delivery approach might be feasible and efficient in the treatment of MI.
